# Anabolic–androgenic steroids and dietary supplements among resistance trained individuals in western cities of Saudi Arabia

**DOI:** 10.1186/s13102-021-00345-6

**Published:** 2021-09-28

**Authors:** Ameen Mosleh Almohammadi, Anas Mohammed Edriss, Turki Talal Enani

**Affiliations:** Department of Pharmacy Practice, Faculty of Pharmacy, King Abdulaziz University, P.O. Box 80260, Jeddah, 21589 Saudi Arabia

**Keywords:** Anabolic–androgenic steroids, Dietary supplements, Hormones, Medical supervision, Saudi Arabia

## Abstract

**Background:**

Anabolic–androgenic steroids (AAS) contain testosterone-like androgens and are used as supplements to improve performance, therapeutic measures, appearance, and muscular development.

**Purpose:**

This study aimed to estimate using anabolic–androgenic steroids (AAS) and good and bad practices about dietary supplements among resistance-trained individuals. It further seeked to determine the use of common drugs and supplements containing anabolic steroids among resistance-trained individuals (who work out at the sports centre) and assess users' knowledge about its side effects.

**Methods:**

A cross-sectional survey was conducted at the sports centres of the western cities of Saudi Arabia. A self-administered questionnaire was used to collect data from 120 male resistance-trained individuals.

**Results:**

The majority of the participants (80%) reported that they had not used any hormonal bodybuilding supplement last year, while 20% said they had used such hormonal supplements. Approximately half (52.5%) of participants reported that they always used dietary supplements. A total of 44.2% of participants possessed inadequate knowledge of these products. The main reason behind the use of hormones and supplements was to increase muscle mass.

**Conclusions:**

A minority of resistance-trained individuals in the studied population frequently misused AAS. However, the results cannot be generalised to the whole of Saudi Arabia. AAS consumption can be reduced by enhancing the level of awareness and knowledge of potential adverse health outcomes.

## Background

Anabolic-androgenic steroids (AAS) are synthetic derivatives of a masculine hormone called testosterone; they are frequently used for muscular development and improvement in performance, appearance and therapeutic measures [1]. Anabolic or “muscle-building” effects of these products help increase muscle mass and decrease body fat [2]. They also help in the development of secondary male sexual features such as deep voice, production of hairs on face and body, increased muscle mass, and can enhance aggression [3]. There are some adverse effects associated with the use of these steroids; among these, some are irreversible [4]. Since the 1950s, the use of anabolic steroids by athletes to improve their performance have been common practice; however, the use of steroids in other segments of society is also increasing at an alarming rate [5]. AAS has been linked with multiple organ failure and myocardial infarction in young athletes [6, 7]. The sharing of the needle to inject the steroids also entails the growing risk of the human immunodeficiency virus (HIV) infection, which is a cause of great concern for the community health [8].

In recent years, the increasing interest of young adults in bodybuilding and body fitness had led to the increased consumption of these steroids mainly because of a lack of awareness regarding the adverse effects of AAS on health [9–11]. Illegal use of doping agents is also prohibited in Saudi Arabia and in 2004 an independent national anti-doping organization called Anti-Doping Committee was established in Saudi Arabia for regulating and monitoring the use of doping substances [12]. Despite the regulations, according to a survey, the prevalence of using AAS is high in bodybuilders regularly visiting sports centres in the Jazan region of Saudi Arabia [13]. In Riyadh, there was a culture of ignorance regarding its side effects and promotion of AAS among fellow athletes [14]. Hearne et al. [15], in their systematic review, posit that these drugs are mostly produced and sourced illicitly and are illegally available in gym settings. Hence, despite the regulations and laws, the illegal availability of steroids to young resistance-trained individuals may have enhanced its usage.

Proteins, vitamins, and creatine are the major non-hormonal food supplements [16]. Protein supplements when combined with resistance training proved beneficial in enhancing fat fee mass and strength in healthy individuals [17]. The study by Phillips [18] suggests beneficial effects of dietary protein supplements, especially in adults and older adults. Similarly, better performance among athletes has been observed with the use of protein and other dietary supplements that help athletes meet their nutritional needs and gain muscle mass [19]. Therefore, optimal dietary supplements and protein are recommended and considered good practice in athletes. Alternatively, the unavailability of nutritionists in sports centres and physical strength sports instructors encourage novice members to use food supplements in excess [20]. According to a study on adolescent athelets regarding protein supplements, it was found that they lack information about its safe purchasing and usage, which makes it important to educate coaches, parents and future athletes regarding sports nutrition [21].

The prevalence of the use of AAS and other prohibited substances among resistance-trained individuals and athletes has been assessed by different studies for particular regions in Saudi Arabia [13, 14, 22–24]. It has been observed that the use of AAS in Saudi athletes is higher despite the growing awareness of the harmful effects of AAS [22]. Al-Shammari et al. [25, 25, 25]. Jabari et al. [14] observed that Saudi athletes either lack knowledge about the effects of AAS or ignore the side effects and keep using them to improve their physical body shape and performance. This evidence suggests that lack of awareness or neglect might be the major causes of increased usage of AAS.

With respect to the above-mentioned issues with the use of AAS and benefits associated with dietary substances. There is a need to understand the prevalence of use of anabolic steroid hormones and practices related to food supplements and evaluate the awareness level of the people living in the West-coast region of Saudi Arabia regarding the effects of AAS and food supplements. Therefore, the aim of this study was to identify common anabolic steroid drugs and good and bad practice in relation to food supplements used by Saudi resistance-trained individuals; evaluate the ratio of male resistance-trained individuals who use anabolic steroid hormone and identify reasons, side-effects and economic loss associated with the use of anabolic steroid hormone and dietary supplements. Also, the practice of AAS and supplement use was assessed based on age and educational level. This study will help in identifying and understanding the causes and effects of misuse of AAS. Furthermore, recommendations were suggested to help increase awareness and decrease the misuse of AAS among resistance-trained individuals.

## Methods

### Design and setting

A descriptive study design was used to collect data with the help of a specifically designed questionnaire in the Arabic language, which was developed for this study. The study was conducted in three different cities of Saudi Arabia: Taif, Jeddah, and Makkah, between March to July 2019.

### Sampling

A random sampling approach was used to recruit male resistance-trained individuals from sports centres located in the aforementioned cities. Following were the inclusion criteria: young adult male athletes (aged between 18 and 40 years); using sports centres at least in the last six months.

TrainAway website (https://www.trainaway.fit/) was used to find the total number of sports centres located in the three cities. The city was then mapped to find the unregistered establishments. The urban areas of all the three cities were divided into different census tracts, and agents, with key information of their respective work areas, were deployed to seek and identify the establishments not found in the website records. The first contact was made with sports centres to gather fundamental information and present the aims of the research, which was based on scheduled interviews with professionals identified by the sports centres. Regarding sports centres, two were identified in Taif; eight in Jeddah and eight in Makkah.

For sample size estimation, a statistical power analysis was performed using GPower software. With a power of 0.80 and alpha of 0.05, the projected sample size needed for the 0.5 effect size (medium) is approximately 100. Thus our targeted sample size of 144 is more than adequate for achieving the objectives of this study. Total 144 participants were asked to participate in this study. The resistance-trained individuals who could not be contacted even after three attempts of contacting them were considered to have declined to participate in the study. After the refusal of twenty-four participants, 120 participants completed the face-to-face questionnaire. Fifty participants were recruited from the sports centres of Makkah (n = 50) and Jeddah (n = 50), while 20 participants were recruited from the sports centres of Taif.

### Data collection

Ethical approval of the Institutional Review Board (IRB) was obtained from the Research Ethics Board of King Abdulaziz University (KAU) (IRB 15–365). Eligible sports athletes were invited to participate in this study after giving them a full explanation of the study objectives. Furthermore, an assessment sheet was provided to participants, which states the study objectives and inclusion/exclusion criteria to both confirm the eligibility of the participants and to provide detailed information regarding this study. In addition to the contact information of the researchers, a consent form was also attached with the questionnaire. All participants also provided written consent of their participation, and they were also assured that data would be kept confidential and that their participation was completely voluntary, and they could withdraw from the research at their free will.

### Instrument

A questionnaire was distributed among the participants. It comprised of three sections: the first section was designed to gain the demographic information of the participants (age, education level, city, and monthly income); the second section assessed person-related product questions (such as awareness regarding side effects of hormones, reasons for using supplements, source of getting hormones and experience of any side effect); and the third section assessed product-related questions (such as which supplements were being used, cost of hormones and supplements used, and route of administration). The questionnaire was originally developed in Arabic to facilitate participants in answering the questions and, afterwards, it was translated into the English language to facilitate participants for easy comprehension. Participants were asked to respond freely about the food supplements they use. Participants were also given an opportunity to answer the items of the questionnaire more than once. For instance, questions related to types of dietary supplements, reasons behind their use, and their sources needed more than one answer. For the same reason, the option of “All types” was made available, which helped participants who used all types of dietary supplements or their sources.

### Validity and reliability

After the measurement of the content validity by a panel of experts and formal approval of the questionnaire, a pilot study was conducted to evaluate the reliability of the questionnaire. Ten athletes were recruited for the pilot study as volunteers to estimate the reliability of the questionnaire. The participants were asked questions regarding the understanding, clarity, and readability of the questionnaire. In addition, they were asked to provide additional comments regarding the possible improvement in the instrument. One of the substantial comments suggested that personal data should not be collected in order to increase the response rate of the participants. They eventually reported that the questionnaire was easy and clear; however, the data from the pilot study were not included in the current study. Cronbach alpha was used to estimate the reliability of the questionnaire where the values were more than 0.7 and therefore were accepted.

### Data analysis

Statistical Package for Social Sciences (SPSS) version 21 (IBM, Armonk, NY United States) was used to analyze the data. Frequencies and descriptive statistics were used to describe all three sections of the questionnaire. Pearson Chi Squared Test was used to examine if there was a difference in the distribution of categorical variables for the populations (i.e. age group and education group) when using AAS and supplements. A p-value of less than 0.05 was considered significant.

## Results

Out of 144 participants, 120 forms were duly filled, making the response rate 83.3%. Among 120 resistance-trained individuals, the average age was 27.78 years (SD = 6.82), where 46.67% of participants possessed a Bachelor's degree and 32.50% participants were diploma holders. The income range of nearly half participants was between 1000–5999 riyals ($266.29-$1597.47). The majority of the participants (80.00%) reported that they had not used any hormonal product for bodybuilding in the last year, while 20.00% reported they had used the hormonal product. When asked about the use of dietary supplements, 25.00% of participants revealed that they had not used any dietary supplements, while half (53.33%) participants reported their regular usage (Table 1).

**Table 1:** Demographics

Demographics	N (n = 120)	%
Age [mean (SD)]	27.78 (± 6.82)	23.15
*Education*		
High school	39	32.50
Diploma	22	18.33
Bachelor	56	46.67
Postgraduate	3	2.50
*Origin*		
Makkah	50	41.67
Taif	20	16.67
Jeddah	50	41.67
*Monthly income*		
< 1000 (< $266.29)	26	21.67
1000–5999 ($266.29-$1597.47)	57	47.50
6000–9999 ($1597.74-$2662.63)	25	20.83
> 10,000 (> $2662.90)	12	10.00
*Use of anabolic hormones*		
Currently using it	24	20.00
Never	96	80.00
*Use of dietary supplements*		
Regularly use	64	53.33
Rarely	26	21.67
Never	30	25.00

A total of 35.83 participants revealed that they were aware of the side effects of AAS, while only 20.00% indicated that they were not aware of their side effects, and about 44.17% of participants had low-level information about AAS (Table 2). Eighty-five per cent of the participants had not used hormones, while 5.00% of participants used Dianabol and 4.17% used Deca Durabolin as a principal hormone. Whereas 3 (2.50%) participants did not reply.

**Table 2:** Awareness about indication and side effects and hormones used

Side effects	N (n = 120)	%
Not at all	24	20.00
Low knowledge	53	44.17
High knowledge	43	35.83
*Hormones used*		
Did not reply	3	2.50
No	102	85.00
Deca durabolin	5	4.17
Dianabol	6	5.00
Testerone cypionate	2	1.67
Deca durabolin + testerone cypionate	1	0.83
Used all these product	1	0.83

According to their lifestyle, the protein was the most used dietary supplement: 25.00% alone and 16.67% in combination with glutamine or vitamin (Table 3). Forty per cent of the participants revealed that they had been using dietary supplements to increase muscle mass, 16.67% used them to speed up muscle growth, and 17.50% used them for both reasons. In comparison, 24.17% of participants revealed that they had no reason to use dietary supplements (Table 3).

**Table 3:** Types of dietary supplements used and reasons of using

Types	N (n = 120)	%
No	33	27.50
Protein	30	25.00
Glutamine	3	2.50
Vitamin	12	10.00
Protein + Glutamine	8	6.67
Protein + Vitamin	12	10.00
Glutamine + Vitamin	1	0.83
All types	21	17.50
*Reasons*		
No reason to use	29	24.17
Increase muscle	48	40.00
Speed muscle growth	20	16.67
Increase muscle + speed muscle growth	21	17.50
Increase + mimic	1	0.83
All types	1	0.83

Table 4 shows the financial cost of anabolic hormone and dietary supplement use. The majority of the participants reported that they had not used anabolic hormones. While 4.17% of participants reported that anabolic hormones cost them 1000–2999 Saudi riyals and 3.33% of participants reported that supplements cost them 500–999 Saudi riyals.

**Table 4:** Financial cost of anabolic hormones and dietary supplements

Anabolic hormones	N (n = 120)	%
No cost	105	87.50
< 500 (< $133.14)	2	1.67
500–999 ($133.14-$266.02)	4	3.33
1000–2999 ($266.29-$798.60)	5	4.17
> 3000 (> $798.87)	4	3.33
*Dietary supplements*		
No cost	15	12.50
< 500 (< $133.14)	43	35.83
500–999 ($133.14-$266.02)	45	37.50
1000–2999 ($266.29-$798.60)	15	12.50
> 3000 (> $798.87)	2	1.67

Table 5 shows the sources from which participants get their products. A total of 24.17% of participants revealed that they did not use any source since they were non-users, while 19.17% of participants got products from the internet, 14.17% from the coach and 13.33% obtained them from their friends.

**Table 5:** Sources of getting products

Responses	N (n = 120)	%
No	29	24.17
Pharmacy	15	12.50
Friends	16	13.33
Coach	17	14.17
Internet	23	19.17
More than one sources (Pharmacy, friends, coach, internet)	18	15.00
All	2	1.67

Oral intake was the most common route of taking the supplements (66.67%) in the participants. A minority (10.83%) of participants used both oral and injection route of taking the supplements. No side effects were experienced by 90.83% of the participants while using these products (Table 6).

**Table 6:** Route of administration and side effects of anabolic hormones

Route	N (n = 120)	%
No	24	20.00
Injection	3	2.50
Oral	80	66.67
Injection + oral	13	10.83
*Side effects*		
Weakness	5	4.17
Headache	4	3.33
Increase hair	2	1.67
Nothing	109	90.83

Table 7 and 8 assessed the association between age and educational status with the usage of AAs and dietary supplements, respectively. No significant association with education was present. However, the majority of students, i.e. 15 (62.50%) among the AAS users, were significantly within the 18–25 years of age (*P* = 0.04). Similarly, 36 (56.25%) users among the daily users of the dietary supplement were significantly within the 18–25 years of age (*P* = 0.031).

**Table 7:** Chi square test for association between demographics and AAS usage

	Use of AAS		*P* value
	Currently using it N (%)	Never N (%)	
*Age*			
18 ≥ to 25 years	15 (26.31)	42 (73.68)	0.04
> 25 to 32 years	7 (17.07)	34 (82.93)	
> 32 years	2 (9.09)	20 (90.91)	
*Education*			
High school	7 (17.95)	32 (82.05)	0.18
Diploma	5 (22.73)	17 (77.27)	
Bachelor	12 (21.43)	44 (78.57)	
Postgraduate	0 (0.00)	3 (100.00)

**Table 8:** Chi square test for association between demographics and dietary supplement usage

Variables	Use of dietary supplements			
	Regularly N (%)	Rarely N (%)	Never N (%)	
*Age*				
18 ≥ to 25 years	36 (63.15)	9 (15.79)	12 (21.05)	0.031
> 25 to 32 years	16 (39.02)	10 (24.39)	15 (36.58)	
> 32 years	12 (54.54)	7 (31.81)	3 (13.64)	
*Education*				
High school	22 (56.41)	4 (10.25)	13 (33.33)	0.052
Diploma	9 (40.91)	8 (36.36)	5 (22.73)	
Bachelor	33 (58.93)	14 (25.00)	9 (16.07)	
Postgraduate	0 (0.00)	0 (0.00)	3 (100.00)	

## Discussion

To the author's knowledge, this is the first study which was conducted in Saudi Arabia that aimed at discovering the use of anabolic hormones and dietary supplements in resistance-trained individuals of three major western cities who visit bodybuilding sports centres on a daily basis. The study findings revealed that dietary supplements were used more frequently than anabolic hormones. Most of the participants (44.2%) have a low level of knowledge about AAS and their side effects. Although the percentage of AAS use was not high, it was higher in younger individuals than older individuals. In contrast, educational status was not related to the usage of AAS or supplements.

The findings of the previous studies [22, 24, 25] reported high usage of AAS in bodybuilders and low-level knowledge regarding its possible harms. It has been observed that appropriate knowledge and awareness are crucial for discouraging athletes from the use of these substances [26–29]. This new body image is leading to psychological body disorders and increasing the usage of AAS in men [29, 22, 8, 30

The highest percentage of AAS users and dietary supplement users were among the 18–25 years of age group, whereas education was not significantly associated with these demographic factors. It appears that younger individuals are more willing to take steroids and dietary supplements than older individuals. Thus, the suggested implication is to target younger individuals for supplementation education. Similarly, none of the postgraduate reported using AAS or dietary supplement, which suggests that higher education might have been associated with avoidance of AAS. However, the observed pattern posits a need to provide awareness regarding the good and bad practice of dietary supplement and precautions needed to be taken with AAS, as usage or non-usage of one does not mandate usage or non-usage of other.

The use of self-efficacy might be considered to prevent AAS usage in young individuals [31]. Self-efficacy is a significant cognitive factor that is behaviour-specific and contributes to the application of healthy behaviours [32^33^,

The main limitation of this study was that it was conducted via a questionnaire-based interview which can cause recall bias. Furthermore, female resistance-trained individuals were not included in this study on account of cultural barriers such as less participation of women in sports or outdoor activities. Due to the lack of female sports centres in the western region, the findings of this study suggest future researchers conduct a similar study based on female resistance-trained individuals. The cross-sectional design used in this study prevented the identification of causality between independent and outcome variables associated with the use of AAS and knowledge and awareness of the health risks. The findings of this study are not generalizable to all regions of Saudi Arabia, as the data was collected from three western regions only, and the study had a small sample size. Therefore, experimental and longitudinal studies are required for examining the effectiveness of interventions in order to prevent the use of AAS in Saudi resistance-trained individuals.

## Conclusion

The study findings revealed that in Saudi Arabia, the percentage of AAS use was not high, but it was higher in younger individuals than older individuals. The main reason identified by the users of AAs was to improve their physical body image and to achieve athletic objectives. Further, they do not have sufficient knowledge regarding the negative effects of the excessive use of this drug. Therefore, it is imperative to educate the current and future resistance-trained individuals of the community, especially the young trainers about the adverse effects of AAS abuse. Counselling regarding body image is recommended to be made available for resistance-trained individuals. The Saudi Food and Drug Authority should carefully examine the illicit manufacturing and distribution of AAS and other harmful supplements for safer usage by consumers.

## Data Availability

The datasets used and analyzed during the current study are available from the corresponding author on reasonable request.

## Abbreviations

AAS: Anabolic–androgenic steroids
HIV: Human immunodeficiency virus
IRB: Institutional review board
KAU: King Abdulaziz university
MoH: Ministry of health
SD: Standard deviation
SPSS: Statistical package for social sciences
